# Atomistic Insights into Impact-Induced Energy Release and Deformation of Core–Shell-Structured Ni/Al Nanoparticle in an Oxygen Environment

**DOI:** 10.3390/ma17164034

**Published:** 2024-08-14

**Authors:** Kexin Zhu, Yifan Xie, Jian-Li Shao, Pengwan Chen

**Affiliations:** 1 State Key Laboratory of Explosion Science and Safety Protection, Beijing Institute of Technology, Beijing 100081, China; 15937607147@163.com (K.Z.); 19801239680@163.com (Y.X.); pwchen@bit.edu.cn (P.C.); 2 Explosion Protection and Emergency Disposal Technology Engineering Research Center of the Ministry of Education, Beijing 100039, China

**Keywords:** Ni/Al nanoparticle, core–shell structure, impact-induced energy release, molecular dynamic

## Abstract

In actual atmospheric environments, Ni/Al composites subjected to high-velocity impact will undergo both intermetallic reaction and oxidative combustion simultaneously, and the coupling of mechanical and multiple chemical processes leads to extremely complex characteristics of energy release. This work employs ReaxFF molecular dynamics simulations to investigate the impact-induced deformation and energy release of a core–shell-structured Ni/Al nanoparticle in an oxygen environment. It was found that Al directly undergoes fragmentation, while Ni experiences plastic deformation, melting, and fragmentation in sequence as the impact velocity increased. This results in the final morphology of the nanoparticles being an ellipsoidal-clad nanoparticle, spherical Ni/Al melt, and debris cloud. Furthermore, these deformation characteristics are strongly related to the material property of the shell, manifested as Ni shell–Al core particle, being more prone to breakage. Interestingly, the dissociation phenomenon of Ni–Al–O clusters during deformation is observed, which is driven by Ni dissociation and Al oxidation. In addition, the energy release is strongly related to the deformation behavior. When the nanoparticle is not completely broken (Ni undergoes plastic deformation and melting), the energy release comes from the oxidative combustion of Al fragments and the intermetallic reaction driven by atomic mixing. When the nanoparticle is completely broken, the energy release mainly comes from the oxidative combustion of the debris cloud. At the same time, the promoting effect of oxygen concentration on the energy release efficiency is examined. These findings can provide atomic insights into the regulation of impact-induced energy release for reactive intermetallic materials.

## 1. Introduction

Ni/Al nanocomposite is a highly representative reactive intermetallic material that undergoes intermetallic reactions and releases a large amount of heat under impact conditions [1]. However, in actual environments, the energy release behavior is often accompanied by material fragmentation and oxidation combustion processes [2]. Intensive research on impact-induced energy release is a longstanding and challenging endeavor due to the simultaneous issues of material mechanics and multiple reaction processes.

In real-world conditions, Al and Ni metals undergo oxidation reactions, forming oxidation products on their surfaces. Understanding the oxidation mechanisms of these metals can enhance the utilization of Al in propellants, explosives, weapons, and other fields. Qu analyzed the thermal reaction characteristics of nano-aluminum powder in different oxygen–nitrogen mixtures during experiments. It was found that at different pre-treatment temperatures, the surface morphology of aluminum particles showed agglomeration, melting deformation, cracking, and fragmentation [3]. In anaerobic environments, aluminum oxidation in aluminum explosives remains stable, while in aerobic environments, anaerobic reactions cannot be ignored [4]. Risha’s experimental study on the combustion behavior of nano-aluminum and liquid water revealed that combustion behavior is inversely proportional to particle size [5]. At high heating rates (10^6^ K/s or higher), it was first discovered that Al nanoparticles oxidize through a melt dispersion mechanism [6]. Additionally, when aluminum nanoparticles burn under reflected shock wave loading, a short-term strong light emission is observed [7].

Currently, experimental research remains at a phenomenological stage, with a lack of detailed analysis on the mechanism of oxidation reactions. In recent years, molecular dynamics simulations have gradually been used to explore the oxidation mechanism of Al nanoparticles from a microscopic perspective. At low temperatures and low oxygen concentrations, Al nanoparticles exhibit a chain oxidation mode [8], while at high temperatures and high oxygen concentrations, they undergo a micro-explosion-accelerated oxidation mode [9]. Zhang investigated the oxidation mechanism of Al nanoparticles of different sizes in oxygen and identified two distinct oxidation stages: a fast oxidation stage and a slow oxidation stage [10]. Beyond examining the oxidation mechanism of Al nanoparticles in oxygen, studies have shown that Al nanoparticles undergo bag-shaped and oscillating deformation modes when moving at high speed in an inert gas-like helium. This reveals the thermodynamic behavior of Al nanoparticles and highlights various temperature rise effects [11]. In practical applications, the surface of Al nanoparticles is typically covered with an aluminum oxide film, forming core/shell nanoparticles. The oxidation mode of Al nanoparticles with an oxide shell differs from that of pure Al nanoparticles. At low heating rates, Al core/Al_2_O_3_ shell nanoparticles primarily exhibit a diffusion oxidation mechanism [12,13], whereas at high heating rates, they display a melt-dispersion oxidation mode [6,14,15,16]. However, under high-speed flowing oxygen, the oxidation mode of Al core/Al_2_O_3_ shell nanoparticles varies with the oxidant flow rate, showing diffusion oxidation, anisotropic oxidation, and micro-explosion oxidation modes [17,18,19].

The oxidation behavior of Ni nanoparticles has also been widely studied and discussed. During the oxidation process, the rapid outward diffusion of Ni ions leads to the formation of hollow oxide shells [20,21]. Under certain conditions, the oxidation mode of Ni nanoparticles transitions from a diffusion oxidation mechanism to a pseudo-homogeneous reaction [22]. Additionally, during the oxidation of Ni nanoparticles, pores and transverse cracks form [23]. Knez estimated the size-dependent oxidation rate of Ni clusters through experiments and molecular dynamics simulations [24]. Sainju proposed an oxidation theory to analyze the size-dependent oxidation of Ni nanoparticles, finding that the oxidation process consists of the NiO nucleation stage and the Wagner diffusion equilibrium NiO shell thickening stage [25].

Ni/Al composites undergo self-sustaining intermetallic reactions under thermal and impact loading, leading to the formation of intermetallic compounds [26]. Current research focuses on the effects of the initial microstructure of nano-multilayer films on these reactions. The initial microstructure includes pores [27], interface structure [28], and premixed layers [29,30], all of which influence reaction kinetics. The reaction mechanisms and damage behavior of Al and Ni nanoparticles under specific loading conditions have also been explored. Evteev found that the alloying reaction of Al shell–Ni core nanoparticles under heating begins with the mutual diffusion of the Ni core and Al shell. This diffusion drives the formation of an amorphous Fcc Ni–Al phase at the interface, eventually rendering the entire shell amorphous [31]. The reaction of Al shell–Ni core nanoparticles forms a Ni–Al layer at the interface, which hinders atomic diffusion and slows down the alloying reaction [32]. When Ni and Al nanoparticles collide, in addition to alloying reactions, damage such as delamination and fragmentation also occurs [33].

Experimental results show that when energetic, structural Ni/Al materials collide in air, the oxidation reaction often predominates. Adding W to energetic, structural Al/Ni materials reveals that the Al–Ni metal reaction and oxidation reaction can trigger a metal reaction between W and Al and Ni, thereby increasing the temperature [34,35]. When CuO is added, the oxidation reaction of Al and Ni is accelerated [36]. These studies indicate that the reaction of Ni/Al core–shell-structured nanoparticles under real-world conditions usually involves oxygen. However, the reaction mechanism of Ni/Al core–shell nanoparticles colliding in oxygen remains unclear.

This work employs ReaxFF molecular dynamics simulations to investigate the deformation and energy release mechanisms of core/shell-structured Ni–Al nanoparticles under high-velocity impact in an oxygen environment. The paper is divided into four sections. The first section provides the research background. The second section introduces the relevant computational details. The third section discusses and analyzes the results. The fourth section presents the research conclusions.

## 2. Materials and Methods

This paper utilizes the Large Scale Atomic/Molecular Massively Parallel Simulator (LAMMPS 64-bit 23Jun2022-MPI) for simulations [37], employing the ReaxFF potential developed by Purja Pun and Mishin [38]. This potential function can describe the breaking and formation of bonds during chemical reactions and is widely used in fields such as metal oxidation reactions [39,40,41,42,43,44,45]. The specific Reaxff potential function parameters used in this paper are those developed by the theoretical team of Adri C.T. van Duin [39]. The simulation in this paper involves the intermetallic reaction between Ni and Al, as well as the oxidation reaction of Ni and Al. The force field selected in this paper has unique advantages in the breaking and formation of metallic bonds and oxidation bonds [46]. Therefore, the force field in this paper is suitable for simulating the collision-induced reaction behavior of Ni/Al nanoparticles.

**Figure 1:**
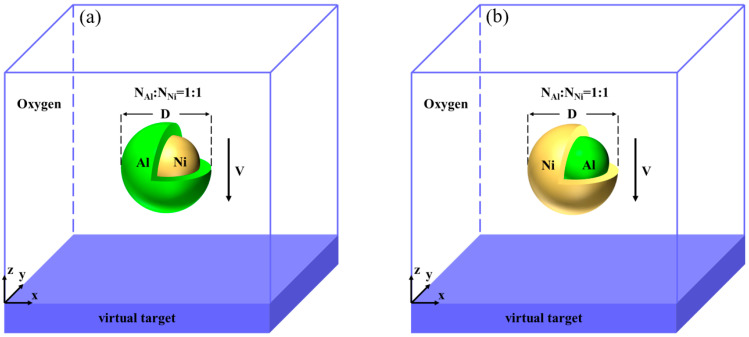
Initial model of nanoparticles impacting the target plate in an oxygen atmosphere. The impact direction is along the Z-axis. (**a**) Al shell–Ni core nanoparticles impacting the target plate. (**b**) Ni shell–Al core nanoparticles impacting the target plate.

Figure 1 illustrates a schematic of core–shell-structured Ni/Al nanoparticles impacting a target plate in an oxygen environment. The target plate is virtual and does not represent a physical object. When the nanoparticles impact this target plate, they experience complete elastic deformation. Two models are considered: (a) Al shell–Ni core nanoparticles impacting the target plate, and (b) Ni shell–Al core nanoparticles impacting the target plate. In the figure, yellow represents the Ni atoms, green represents the Al atoms, and the oxygen molecules are distributed throughout the space. Both core–shell nanoparticles have a diameter of 20 nm. The atomic ratio of Al to Ni in the core/shell nanoparticles is maintained 1:1, which corresponds to the atomic ratio of the NiAl intermetallic phase. This intermetallic phase is a typical component of the Ni–Al alloy and is known for its high stability. The dimensions of the simulation box are 320 nm in the x and y directions and 40 nm in the z direction. In order to explore the collision reaction mechanism of the Ni/Al nanoparticles in a high-temperature and high-pressure gas environment, the density of oxygen in the box is set to 0.00143, 0.00286, and 0.00429 g/cm^3^ according to relevant literature [47]. The gas pressure of oxygen varies with density: at 0.00143 g/cm^3^, it is 0.8 atm; at 0.00286 g/cm^3^, it is 16 atm; and at 0.00429 g/cm^3^, it is 24 atm. The velocities considered are 1, 1.5, 2, and 2.5 km/s.

First, the oxygen was relaxed for 10 ps in the NVT ensemble (*T* = 2000 K) to achieve thermodynamic equilibrium. Next, the core–shell structure was relaxed, with Al and Ni atoms allowed to equilibrate for 20 ps in the NVT ensemble (*T* = 300 K). Finally, the core–shell nanoparticles were introduced into the oxygen environment, and impact simulations were conducted in the NVE ensemble with a time step of 0.5 fs. Periodic boundary conditions were applied in the X and Y directions, while fixed boundary conditions were used in the Z direction.

The visualization software used was the open-access tool, OVITO 3.7.11 [48]. Dynamic deformation of the material was analyzed using adaptive nearest neighbor analysis (a-CNA) to track changes in the atomic crystal structure [49]. If a Ni atom is found within a 0.25 nm radius of an Al atom, the Al atoms are classified as part of the intermetallic phase [50,51]. Local temperatures were calculated based on the thermal velocities, determined by subtracting the center of mass velocity within the atom’s spherical range. A three-dimensional bin analysis was employed to assess the overall changes in thermal kinetic energy during the impact [52].

## 3. Results and Discussion

### 3.1. Analysis of Nanoparticle Deformation and Damage

The deformation and damage behavior of nanoparticles are influenced by impact velocity. In this section, the effects of different impact velocities and core–shell structures on the deformation and damage mechanism of nanoparticles were studied by analyzing the atomic morphology and local temperature.

**Figure 2:**
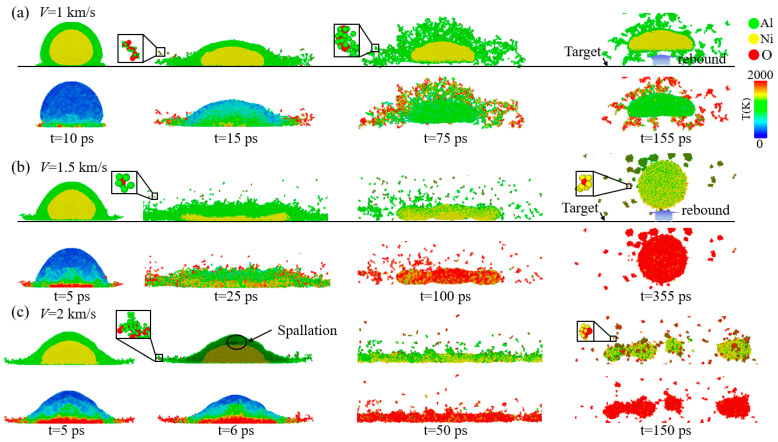
The oxygen concentration is 0.00286 g/cm^3^. The morphology and local temperature evolution of the Al shell–Ni core nanoparticles at different velocities. (**a**) *V* = 1 km/s. (**b**) *V* = 1.5 km/s. (**c**) *V* = 2 km/s.

Figure 2 illustrates the morphology and local temperature evolution of Al shell–Ni core nanoparticles at different velocities, with an oxygen concentration of 0.00286 g/cm^3^. At *V* = 1 km/s, the contact surface temperature rises rapidly at the onset of impact. The Al shell is squeezed and deformed toward both ends, causing the Ni core to shift from a spherical to an elliptical shape. At *t* = 15 ps, the Al shell breaks at both ends, producing numerous Al clusters. During this process, oxygen is adsorbed and reacts to form Al–O clusters. By *t* = 75 ps, the Al shell begins to micro-explode and decompose into smaller clusters due to the rapid increase in local temperature from the heat release of the Al shell and oxygen reaction. This micro-explosion accelerates the oxidation process, a phenomenon frequently mentioned in the Al nanoparticle oxidation literature [9]. The Ni core remains solid throughout, with minimal intermetallic reaction observed on its surface. By *t* = 155 ps, the Al shell is fully decomposed, and the Ni core maintains its solid structure with low intermetallic reaction. Rebound also occurs throughout the deformation and damage process. At *V* = 1.5 km/s, the deformation of the Al shell is similar to that observed at *V* = 1 km/s. However, the Ni core initially compresses and deforms into a flattened shape before contracting into a spherical form, which is more stable and has lower surface energy. During this process, the Ni core melts, intensifying the mixing of Al and Ni, leading to a violent metal–metal reaction and the formation of Ni–O clusters. The Ni core ultimately forms a molten Al–Ni–O cluster with significant Al–Ni mixing, accompanied by rebound. At *V* = 2 km/s, micro-layer cracking damage occurs at the junction of the Al shell and the Ni core due to the formation of a tensile wave that propagates inward after stress reaches the free surface. High impact strength results in rapid breakage of the Al shell and Ni core, generating a large number of oxide clusters.

**Figure 3:**
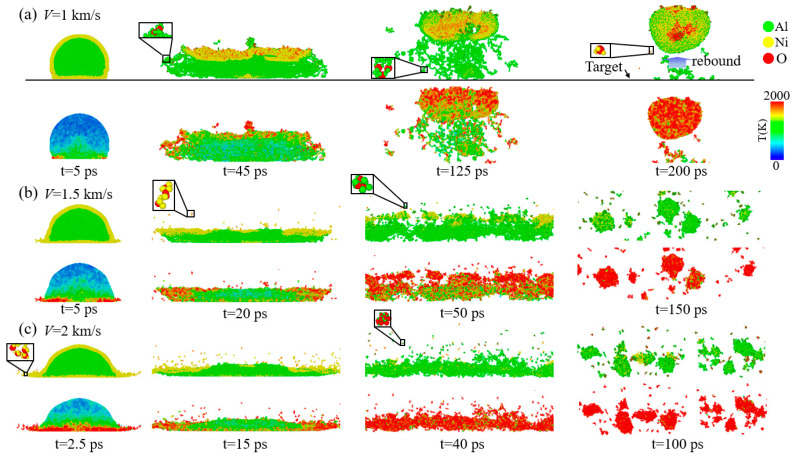
The oxygen concentration is 0.00286 g/cm^3^. The morphology and local temperature evolution of the Ni shell–Al core nanoparticles at different velocities. (**a**) *V* = 1 km/s. (**b**) *V* = 1.5 km/s. (**c**) *V* = 2 km/s.

Figure 3 shows the morphology and local temperature evolution of Ni shell–Al core nanoparticles at different velocities. At *V* = 1 km/s, the Al core rapidly amorphizes at the onset of impact, while the Ni shell at both ends experiences compression and deformation but does not fragment, unlike the Al shell–Ni core nanoparticles. At *t* = 45 ps, the Ni shell–Al core nanoparticles become flattened, with oxygen adsorbing onto the upper end of the Ni shell, leading to end breakage. The internal Al core begins to react with the oxygen, releasing heat and causing a rapid rise in local temperature. Subsequently, the Ni shell starts to curl, and the Al core decomposes into small clusters. By *t* = 200 ps, the Ni shell has curled into a spherical shape, with a high concentration of oxygen atoms in the center, increased mixing of Al and Ni atoms, and deformation accompanied by rebound. When *V* ≥ 1.5 km/s, as impact intensity increases, both the Al core and Ni shell break, generating numerous Al–O and Ni–O clusters. However, the Al shell–Ni core nanoparticles remain intact until the velocity reaches 2 km/s. This indicates that Ni shell–Al core nanoparticles are more prone to breakage and exhibit lower impact strength compared to Al shell–Ni core nanoparticles.

Interestingly, we also observe the dissociation of the Al–Ni–O clusters during the deformation process, which will be analyzed in the following section.

**Figure 4:**
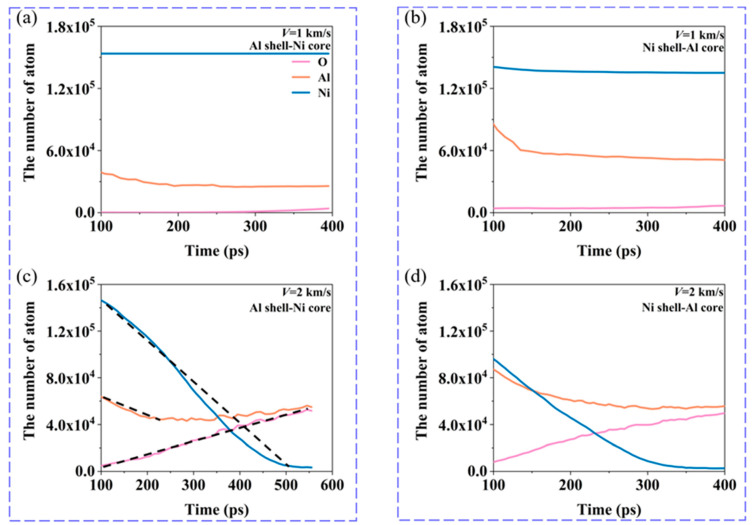
Evolution of the number of different type atoms in Al–Ni–O clusters for nanoparticles at different velocities when oxygen concentration is 0.00286 g/cm^3^. When *V* = 1 km/s, (**a**) Al shell–Ni core nanoparticles and (**b**) Ni shell–Al core nanoparticles. When *V* = 2 km/s, (**c**) Al shell–Ni core nanoparticles. The slope of the black dashed line represents the atomic change rate. (**d**) Ni shell–Al core nanoparticles.

Figure 4 shows the curves of the number of each type of atom (Al, Ni, and O) over time in all Al–Ni–O clusters containing aluminum, nickel, and oxygen atoms at *V* = 2 km/s and *V* = 1 km/s. The slope of the black dashed line in Figure 4c represents the atomic change rate. The slope is calculated by Δ*N*/Δ*t* (Δ*N* is the change in the number of atoms (Al, Ni, O) in the Al–Ni–O cluster when it begins to change to an almost stable value. Δ*t* is the time change for that process.) The rate of atomic change in other cases is also calculated in this way. The changes in the rate of atomic change under different loading conditions are discussed in the following chapters. In the figure, the starting time is 100 ps, by which point the nanoparticles have been completely broken after impact, so the effect of impact on the dissociation of the Al–Ni–O clusters is not considered. At *V* = 1 km/s, the number of Ni atoms in the Al–Ni–O clusters of both nanoparticle structures remains relatively constant, while the number of Al atoms decreases. The reduction in Al atoms is more pronounced in the Ni shell–Al core nanoparticles. At this velocity, the dissociation mechanism primarily involves a decrease in Al atoms, with a relatively low degree of dissociation. At *V* = 2 km/s, for the Al shell–Ni core nanoparticles, at *t* = 100 ps, the number of Ni atoms in the Al–Ni–O clusters is highest, followed by Al atoms, with oxygen atoms being the fewest. As the Al–Ni–O clusters dissociate, the number of Ni atoms gradually decreases, Al atoms decrease to some extent, and the oxygen content in the clusters increases. Over time, the number of Al and O atoms in the Al–Ni–O clusters becomes more comparable, and the total number of atoms in the clusters decreases. For the Ni shell–Al core nanoparticles, at *t* = 100 ps, the atomic composition of the Al–Ni–O clusters differs from that of the Al shell–Ni core nanoparticles. Finally, the number of Al and Ni atoms is roughly equal, and the number of O atoms is relatively low. As the Al–Ni–O clusters dissociate, the number of Ni atoms drops sharply, and the oxygen content of the clusters increases. This dissociation mechanism is similar to that observed in the Al shell–Ni core nanoparticles, but the time required for complete dissociation is significantly reduced. In both structures, the dissociation mechanism is primarily driven by the reduction in Ni atoms, accompanied by an increase in the oxygen content. Ultimately, the number of Ni atoms in the Al–Ni–O clusters is minimized, with the ratio of Al to O atoms approaching 1:1.

**Figure 5:**
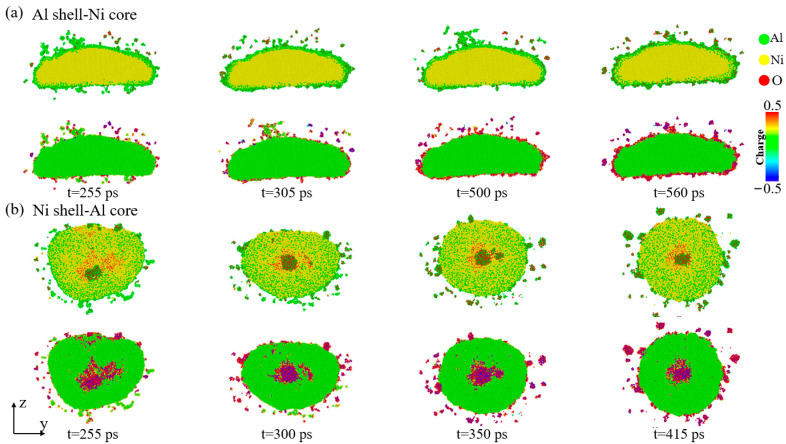
Morphological evolution and corresponding charge maps of Al–Ni–O clusters during dissociation at *V* = 1 km/s when oxygen concentration is 0.00286 g/cm^3^. (**a**) Al shell–Ni core nanoparticle. (**b**) Ni shell–Al core nanoparticle.

Figure 5 illustrates the atomic morphology and charge distribution of the dissociation process of the Al–Ni–O clusters in the two types of nanoparticles at *V* = 1 km/s. As shown in Figure 2, the Al shell–Ni core nanoparticles are not fully broken at this stage. The Al–Ni–O clusters are primarily composed of Ni cores. Figure 5a shows that the surface of the Al–Ni–O clusters is covered with uneven Al–O compounds. Over time, the surface of these clusters begins to dissociate, resulting in the formation of Al clusters and Al–O clusters. The increasing oxygen content on the surface leads to the formation of a uniform aluminum oxide film, which protects the Ni core from dissociation. For the Al–Ni–O clusters in the Ni shell–Al core nanoparticles, smaller and less stable aluminum oxide clusters form on the surface. At high temperatures, a small number of Al and Ni atoms break away from the surface of the Al–Ni–O cluster. Additionally, the Al and Ni atoms within the Al–Ni–O clusters become highly mixed. The shape of the cluster gradually changes from an ellipsoid to a spherical form, which is relatively stable. In summary, when the nanoparticles are not completely broken, the dissociation mode of the Al–Ni–O clusters is primarily characterized by a reduction in Al atoms, which are removed from the clusters through the formation of aluminum oxide clusters.

**Figure 6:**
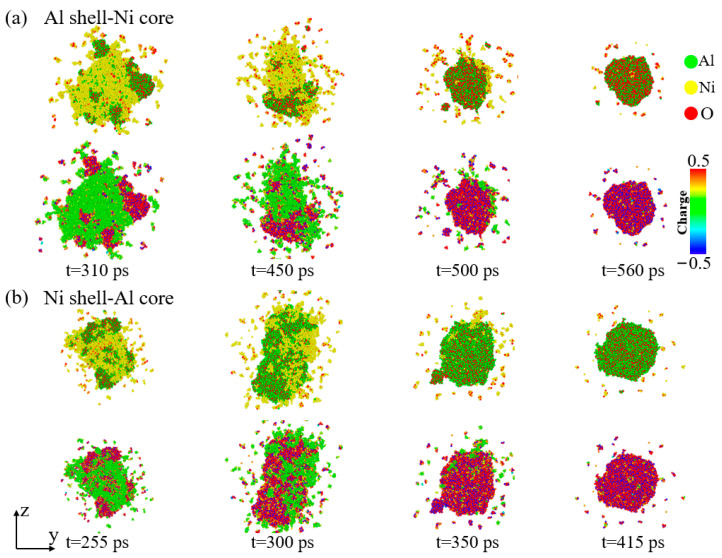
Morphological evolution and corresponding charge maps of Al–Ni–O clusters during dissociation at *V* = 2 km/s when oxygen concentration is 0.00286 g/cm^3^. (**a**) Al shell–Ni core nanoparticle. (**b**) Ni shell–Al core nanoparticle.

Figure 6 illustrates the atomic morphology and charge distribution during the dissociation process of the Al–Ni–O clusters in the two types of nanoparticles at *V* = 2 km/s. For the Al–Ni–O clusters in the Al shell–Ni core nanoparticles, from *t* = 310 ps to 450 ps, the Ni atoms begin to gradually separate from the clusters, exposing the Al–O clusters that are previously wrapped by Ni atoms. At *t* = 500 ps, most of the Ni atoms on the surface of the Al–Ni–O cluster have dissociated, exposing the inner Al–O cluster. By *t* = 560 ps, the majority of Ni atoms have dissociated from the clusters, leaving only a few Ni atoms on the surface. The Al–Ni–O clusters take on a spherical shape as they evolve. The charge distribution diagram shows that most of the Al in the clusters reacts chemically with oxygen to form stable oxides, while the Ni atoms do not react with oxygen and are less stable. Under high temperatures, Ni dissociates more readily than Al oxides. Consequently, the final composition of the Al–Ni–O clusters is primarily Al and O. The dissociation time of the Al–Ni–O clusters in the Ni shell–Al core nanoparticles is shorter compared to the Al shell–Ni core nanoparticles.

**Figure 7:**
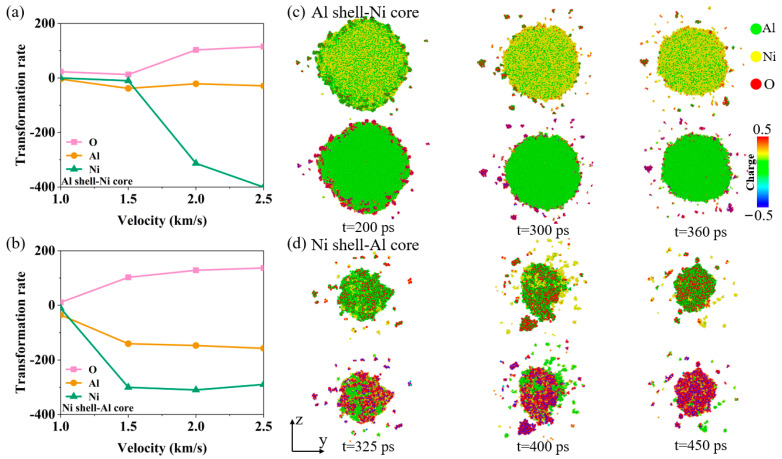
When the oxygen concentration is 0.00286 g/cm^3^: (**a**) Changes in the atomic change rate of the Al–Ni–O clusters for Al shell–Ni core nanoparticles at different velocities. (**b**) Changes in the atomic change rate of the Al–Ni–O clusters of Ni shell–Al core nanoparticles at different velocities. (**c**) Morphological evolution and charge distribution of the Al–Ni–O clusters during dissociation for Al shell–Ni core nanoparticles at *V* = 1.5 km/s. (**d**) Morphological evolution and charge distribution of the Al–Ni–O clusters during dissociation for Ni shell–Al core nanoparticles at *V* = 1.5 km/s.

The above studies show that the Al–Ni–O clusters have different dissociation mechanisms. Figure 7 examines the impact of varying velocities on the rate of change in the number of Al, Ni, and O atoms within the Al–Ni–O clusters for both nanoparticle structures. The changes in the atomic change rate in Figure 7 refer to the slope of the curves representing the total number of each type of atom over time in the Al–Ni–O clusters. For the Al shell–Ni core nanoparticles, complete breakup does not occur at 1 km/s and 1.5 km/s. At these velocities, the dissociation of the Al–Ni–O clusters primarily involves a reduction in Al atoms, with a slight increase in oxygen atoms. When the velocity reaches 2 km/s or higher, the nanoparticles fully disintegrate, and the dissociation pattern shifts to a significant reduction in Ni atoms. This reduction rate is much higher compared to the reduction in Al atoms when the nanoparticles are not fully broken, and it further increases with higher velocities. Additionally, the rate of increase in oxygen atoms also significantly rises compared to the rates at 1 km/s and 1.5 km/s, while the rate of reduction in Al atoms approaches zero. For Ni shell–Al core nanoparticles, the nanoparticles do not break completely at 1 km/s. At this velocity, the dissociation mechanism of Al–Ni–O clusters primarily involves a reduction in Al atoms. When the velocity reaches 1.5 km/s or higher, the nanoparticles are fully broken, and the dissociation mode shows a marked reduction in Ni atoms. The reduction rate of Ni atoms increases significantly, with the overall reduction rate becoming less sensitive to further increases in velocity. However, at these conditions, the reduction rate of Al atoms remains high, indicating that the dissociation of the Al–Ni–O clusters is accompanied by a notable reduction in Al atoms.

### 3.2. Energy Release Characteristics

**Figure 8:**
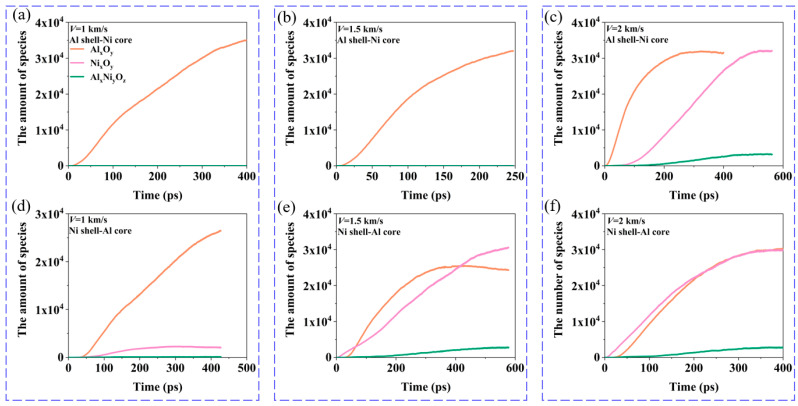
The changes in the number of oxidation clusters for nanoparticles at different velocities when the oxygen concentration is 0.00286 g/cm^3^: (**a**) *V* = 1 km/s, Al shell–Ni core nanoparticle. (**b**) *V* = 1.5 km/s, Al shell–Ni core nanoparticle. (**c**) *V* = 2 km/s, Al shell–Ni core nanoparticle. (**d**) *V* = 1 km/s, Ni shell–Al core nanoparticle. (**e**) *V* = 1.5 km/s, Ni shell–Al core nanoparticle. (**f**) *V* = 2 km/s, Ni shell–Al core nanoparticle.

From Figure 2 and Figure 3, we can see that during the collision, three types of oxidation clusters will react to form Al–O clusters (containing Al and O atoms), Ni–O clusters (containing Ni and O clusters), and Al–Ni–O clusters (containing Al, Ni, O atoms). Therefore, the changes in the number of oxidation clusters in Figure 8 show the evolution of each oxidation cluster over time. Next, we analyze the evolution of oxidation clusters at different velocities, as shown in Figure 8. At *V* = 1 km/s, the oxidation clusters in both Al shell–Ni core and Ni shell–Al core nanoparticles are primarily Al–O clusters. The impact strength is low at this velocity, resulting in incomplete breakup of the Ni core and minimal reaction of internal Ni atoms with oxygen. Consequently, there are few Ni–O and Al–Ni–O clusters. At *V* = 1.5 km/s, the number of Al–O clusters is the highest, while Ni–O and Al–Ni–O clusters are almost non-existent. This is because, similar to *V* = 1 km/s, a significant number of Ni atoms do not react with oxygen. Instead, primarily Al reacts with oxygen, leading to Al–O clusters being predominant. For the Ni shell–Al core nanoparticles, the final oxidation clusters include Al–O, Ni–O, and Al–Ni–O clusters. This occurs because the nanoparticles undergo complete fragmentation during impact, leading to extensive reactions between the Ni and Al clusters with oxygen. The Ni–O clusters form first from the reaction of the Ni shell with oxygen. As the impact progresses, the Al core reacts with oxygen to form the Al–O clusters, followed by the generation of the Al–Ni–O clusters. Eventually, the number of Al–O clusters stabilizes first, with the Ni–O clusters becoming the most numerous, while the Al–Ni–O clusters are the least. At *V* = 2 km/s, the Al–O clusters form initially in the Al shell–Ni core nanoparticles, followed by the Ni–O and Al–Ni–O clusters. After a period of reaction, the Al–O clusters reach a stable value first, followed by the Ni–O and Al–Ni–O clusters. Ultimately, the number of Al–O and Ni–O clusters becomes comparable. For Ni shell–Al core nanoparticles, Ni–O clusters appear first, with Al–O clusters increasing subsequently. Both types of clusters eventually increase and reach a stable value nearly simultaneously.

**Figure 9:**
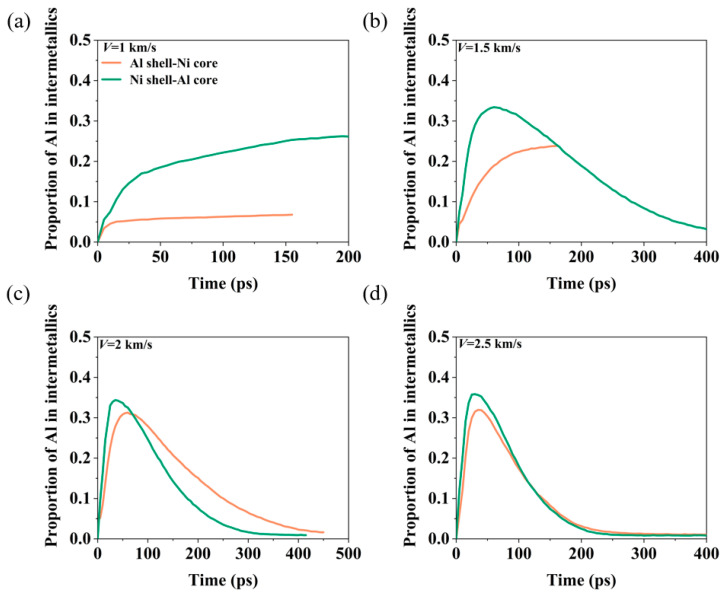
The evolution of the proportion of Al in intermetallics at different velocities when the oxygen concentration is 0.00286 g/cm^3^. (**a**) *V* = 1 km/s. (**b**) *V* = 1.5 km/s. (**c**) *V* = 2 km/s. (**d**) *V* = 2.5 km/s.

Figure 9 illustrates the proportion of Al in intermetallics at different velocities. At *V* = 1 km/s, the Al shell–Ni core nanoparticles undergo a noticeable intermetallic reaction, but the proportion of Al in the intermetallics is higher in the Ni shell–Al core nanoparticles compared to the Al shell–Ni core nanoparticles. Throughout the reaction process, the Ni shell tends to shrink into a spherical nanoparticle with a high degree of Al and Ni atom mixing. In contrast, the Al shell–Ni core nanoparticles only exhibit a reaction on the surface of the Ni core, resulting in a lower degree of Al–Ni mixing. At *V* = 1.5 km/s, the Al shell–Ni core nanoparticles are not completely broken, and their Al–Ni–O clusters form spherical structures with a high degree of Al–Ni mixing and a significant proportion of Al in the intermetallics. For the Ni shell–Al core nanoparticles, the proportion of Al in intermetallics initially increases before gradually decreasing. At *V* ≥ 2 km/s, both types of nanoparticles undergo complete fragmentation. The proportion of Al in intermetallics first increases and then decreases. This trend is linked to the dissociation mechanism of Al–Ni–O clusters. As the nanoparticles break apart, the dissociation rate of Al–Ni–O clusters accelerates, leading to the detachment of Ni atoms from the surface. This separation of Al and Ni atoms results in a reduced proportion of Al in the intermetallics.

**Figure 10:**
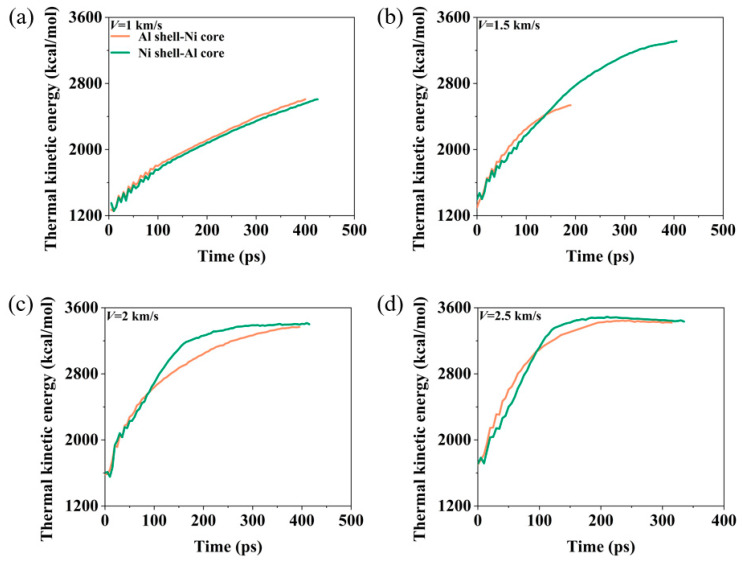
The evolution of thermal kinetic energy of the two nanoparticles at different velocities when the oxygen concentration is 0.00286 g/cm^3^. (**a**) *V* = 1 km/s. (**b**) *V* = 1.5 km/s. (**c**) *V* = 2 km/s. (**d**) *V* = 2.5 km/s.

Figure 10 illustrates the evolution of thermal kinetic energy for nanoparticles at different velocities. At *V* = 1 km/s, the thermal kinetic energy of both types of nanoparticles gradually increases due to the heat released from the reactions. The changes in thermal kinetic energy are relatively similar, with the Al shell–Ni core nanoparticles showing slightly higher values. At *V* = 1.5 km/s, from *t* = 0 to 150 ps, the thermal kinetic energy of both nanoparticle types rapidly rises up due to intermetallic and oxidation reactions, and the thermal kinetic energy values for both types are close during this period. However, as the Ni core of the Al shell–Ni core nanoparticles begins to take on an ellipsoid shape, the Ni core hardly participates in the exothermic reaction, resulting in the thermal kinetic energy quickly reaching the plateau value. Conversely, the Ni shell–Al core nanoparticles undergo violent fragmentation, with the fragments continuing to react and release energy. This causes the thermal kinetic energy to rise further, reaching a plateau at 400 ps. When the velocity is increased to V = 2 km/s or higher, both types of nanoparticles completely break apart. The thermal kinetic energy of the Ni shell–Al core nanoparticles increases more rapidly, indicating a faster reaction rate. Despite this, the final stable thermal kinetic energy values of both types are relatively close, with only minor differences. Additionally, higher velocities lead to shorter times for the complete reaction.

**Figure 11:**
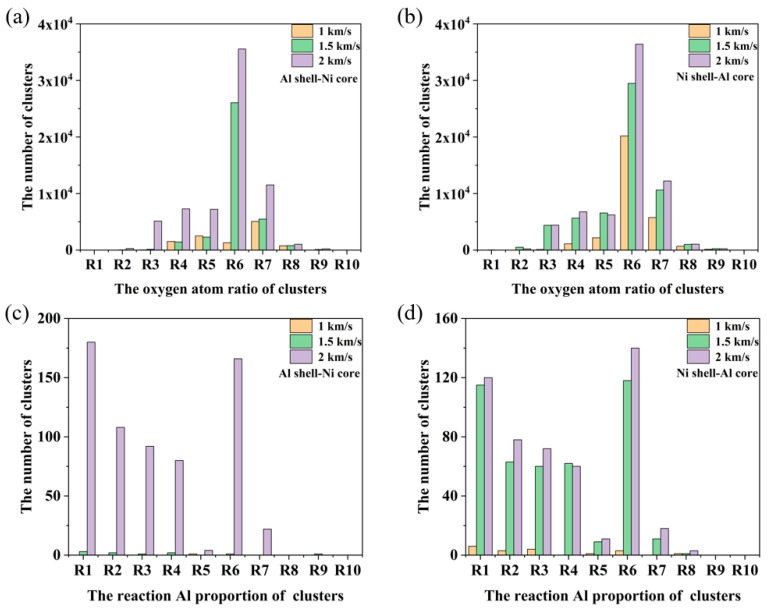
The distribution of different oxygen contents and the proportion of reacted Al in the clusters at varying velocities when the oxygen concentration is 0.00286 g/cm^3^. (**a**) Al shell-Ni core nanoparticles, the distribution of different oxygen contents in the clusters. (**b**) Ni shell-Al core nanoparticles, the distribution of different oxygen contents in the clusters. (**c**) Al shell-Ni core nanoparticles, the distribution of different the proportion of reacted Al in the clusters. (**d**) Ni shell-Al core nanoparticles, the distribution of different the proportion of reacted Al in the clusters.

Figure 11 displays the distribution of different oxygen contents and the proportion of reacted Al in the clusters of nanoparticles with both structures at varying velocities. R1, R2, R3, …, R10 represent the atomic ratio interval of oxygen atoms or reactive Al atoms in the oxidized clusters. R1 range is 0 ≤ R1 < 0.1; R2 range is 0.1 ≤ R2 < 0.2; R3 range is 0.2 ≤ R3 < 0.3, and so on; R10 range is 0.9 ≤ R10 < 1. For the distribution of different oxygen contents in the clusters, the oxygen content in the clusters of Al shell–Ni core nanoparticles is primarily concentrated in ranges R6 (0.5–0.6) and R7 (0.6–0.7). As the velocity increases, the number of clusters in these ranges gradually increases. At velocities of 1 km/s and 1.5 km/s, the number of clusters is relatively small, leading to notable differences in the distribution of clusters with varying oxygen contents. For Ni shell–Al core nanoparticles, the distribution pattern of oxygen content is similar, with the majority of oxygen content concentrated in the 0.5–0.7 range. However, the proportion of clusters undergoing intermetallic reactions is relatively low. The proportion of reacted Al in clusters for both nanoparticle structures is mainly concentrated in ranges R1 and R6 (0–0.1 and 0.5–0.6), while clusters with higher reacted Al in ranges R9 and R10 are minimal, almost non-existent. As the impact velocity increases, the nanoparticles are significantly fragmented, leading to an increase in the number of clusters across all ranges.

### 3.3. The Effect of Oxygen Concentration

Oxygen concentration significantly influences the reaction rate and the changes in cluster size of the nanoparticles during impact. Therefore, this section explores the effect of oxygen concentration on the reaction mechanism.

**Figure 12:**
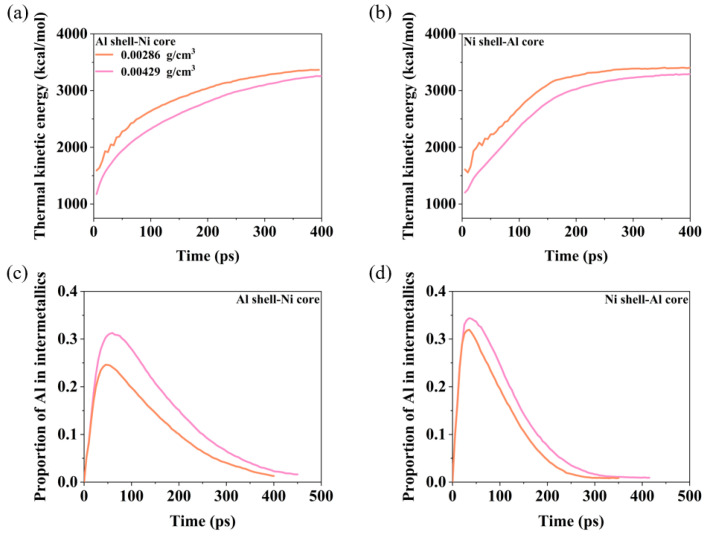
The effect of oxygen concentration on the thermal energy and the proportion of Al in the intermetallics of the two nanoparticles during the reaction process at *V* = 2 km/s. (**a**) The thermal energy evolution of the Al shell–Ni core. (**b**) The thermal energy evolution of the Ni shell–Al core. (**c**) The proportion of Al in the intermetallics of the Al shell–Ni core nanoparticle. (**d**) The proportion of Al in the intermetallics of the Ni shell–Al core nanoparticle.

Figure 12 illustrates the evolution of the proportion of Al in the intermetallics and thermal kinetic energy for nanoparticles with two different structures under varying oxygen concentrations. For both types of nanoparticles, the thermal kinetic energy decreases as the oxygen concentration increases. Initially, upon impact, the intense mixing of Al and Ni atoms leads to a rapid increase in the degree of intermetallic reaction. However, as the Al–Ni–O clusters dissociate, the proportion of Al in intermetallics gradually decreases, resulting in a stabilization of the final thermal kinetic energy. With higher oxygen concentrations, the proportion of Al in intermetallics increases.

**Figure 13:**
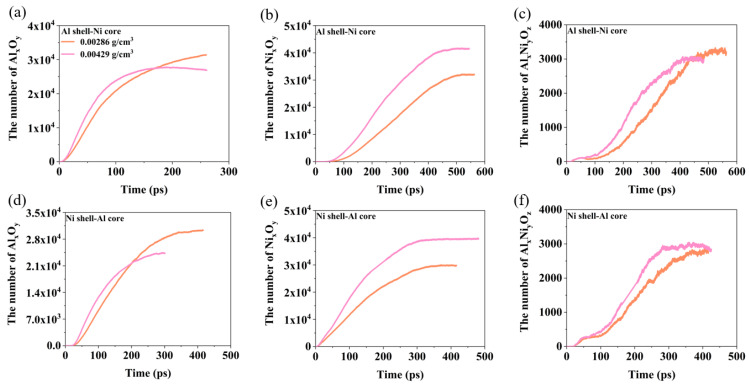
Evolution of the number of oxidation clusters (Al–O, Al–Ni–O, and Ni–O clusters) of two nanoparticles under different oxygen concentrations at *V* = 2 km/s. (**a**) Al shell-Ni core nanoparticles, the evolution of the number of Al-O clusters. (**b**) Al shell-Ni core nanoparticles, the evolution of the number of Ni-O clusters. (**c**) Al shell-Ni core nanoparticles, the evolution of the number of Al-Ni-O clusters. (**d**) Ni shell-Al core nanoparticles, the evolution of the number of Al-O clusters. (**e**) Ni shell-Al core nanoparticles, the evolution of the number of Ni-O clusters. (**f**) Ni shell-Al core nanoparticles, the evolution of the number of Al-Ni-O clusters.

Figure 13 provides an analysis of how the oxygen concentration affects the oxidation clusters. For the Al shell–Ni core nanoparticles, increasing the oxygen concentration accelerates the growth rate of all three types of oxidation clusters and reduces the time required for them to reach a stable value. While the final stable quantities of the Al–O and Al–Ni–O clusters are similar, the oxygen concentration has a more pronounced effect on the number of Ni–O clusters. For the Ni shell–Al core nanoparticles, a similar trend is observed: the growth rate of the oxidation clusters increases and the time to reach a stable value decreases with higher oxygen concentrations. However, the final amount of Al–O clusters decreases as the oxygen concentration rises, whereas the Ni–O clusters increase. The final number of Al–Ni–O clusters remains relatively stable across different oxygen concentrations, with only minor variations.

## 4. Conclusions

This paper employs molecular dynamics (MD) simulations to investigate the impact-induced energy release and deformation of two core–shell-structured Ni/Al nanoparticles in an oxygen environment, with a focus on the effects of impact velocity and oxygen concentration.

The study reveals that Al predominantly undergoes fragmentation during impact, while the deformation mode of Ni varies with impact velocity. At *V* = 1 km/s, the Ni core experiences plastic deformation, eventually forming an ellipsoidal nanoparticle. The Ni shell curls and deforms, resulting in a Ni–Al melt. At *V* = 1.5 km/s, the Ni core melts and subsequently shrinks into a spherical Ni–Al melt, accompanied by a rebound phenomenon. The Ni shell–Al core nanoparticles are completely fragmented. At *V* = 2 km/s, both types of nanoparticles are fully fragmented, forming a debris cloud, with spallation occurring inside the Al shell–Ni core nanoparticles.

Two dissociation mechanisms for Al–Ni–O clusters are identified during nanoparticle deformation. Mechanism 1 involves a small amount of Al atoms on the surface reacting with oxygen to form oxidized clusters, which gradually detach, leading to a decrease in Al atoms. Intermetallic compounds and surface oxide films impede further dissociation. Mechanism 2 involves significant detachment of surface Ni atoms, with a dissociation rate much higher than that of Mechanism 1.

Throughout the impact process, energy is released. When Ni undergoes plastic deformation and melting, the energy is primarily released from the oxidative combustion of Al fragments and intermetallic reactions, with minimal Ni oxidation, resulting in predominantly Al–O clusters. When Ni is completely broken, the energy release is driven by the oxidative combustion of the debris cloud, generating a large number of Al–Ni–O, Ni–O, and Al–O clusters. The proportion of oxygen content in these clusters mainly falls between 0.5 and 0.6. As oxygen concentration increases, the degree of oxidation is enhanced, accelerating the reaction process.

## Data Availability

The raw data supporting the conclusions of this article will be made available by the authors on request.
